# The role of supervision and motivation during exercise on physical and mental health in older adults: a study protocol for a randomized controlled trial (PRO-Training project)

**DOI:** 10.1186/s12877-024-04868-8

**Published:** 2024-03-20

**Authors:** Paola Gómez-Redondo, Pedro L. Valenzuela, Óscar Martínez-de-Quel, Coral Sánchez-Martín, Mónica Cerezo-Arroyo, David Moreno-Manzanaro, Luis M. Alegre, Amelia Guadalupe-Grau, Ignacio Ara, Asier Mañas

**Affiliations:** 1 GENUD Toledo Research Group, Faculty of Sports Sciences, University of Castilla-La Mancha, Avda. Carlos III S/N, 45071 Toledo, Spain; 2 CIBER of Frailty and Healthy Aging, Instituto de Salud Carlos III, Madrid, Spain; 3 Instituto de Investigación Sanitaria de Castilla-La Mancha (IDISCAM), Junta de Comunidades de Castilla-La Mancha (JCCM), Toledo, Spain; 4 Physical Activity and Health Research Group (PaHerg), Research Institute of Hospital, 12 de Octubre (imas12), Madrid, Spain; 5 Department of Systems Biology, University of Alcalá, Madrid, Spain; 6 Didactics of Languages, Arts and Physical Education Department, Faculty of Education, Complutense University of Madrid, 28040 Madrid, Spain; 7 Faculty of Sciences for Physical Activity and Sport (INEF), Polytechnic University of Madrid, 28040 Madrid, Spain; 8 Center UCM-ISCIII for Human Evolution and Behavior, 28029 Madrid, Spain

**Keywords:** Work out, App-based exercise programs, Cognitive-behavioral strategies, Cost-effectiveness, Aging

## Abstract

**Background:**

Although supervised exercise is frequently recommended for older adults, its superiority over unsupervised exercise remains uncertain. Furthermore, whether motivational techniques could help to enhance the effectiveness of the latter remains to be elucidated. The present randomized controlled trial aims to determine the role of supervision and motivational strategies on the safety, adherence, efficacy, and cost-effectiveness of different exercise programs for improving physical and mental health in older adults.

**Methods:**

Participants (*n* = 120, aged 60–75 years) will be randomly allocated into five groups: 1-Control (CON), 2-Supervised exercise without motivational intervention (SUP), 3- Supervised exercise with motivational intervention (SUP +), 4- Unsupervised exercise without motivational intervention (UNSUP) and 5- Unsupervised exercise with motivational intervention (UNSUP +). Over 24 weeks, all exercise groups will participate in a multicomponent exercise program three times/week (performed in group classes at a center for SUP and SUP + , or home without supervision but with the help of a mobile app for UNSUP and UNSUP +), while the CON group will maintain their usual lifestyle. The motivational intervention (for SUP + and UNSUP + groups) will be based on the self-determination theory, including strategies such as phone calls, interactive workshops, motivational messages, informative infographics and videos. Primary outcomes will include safety, adherence, costs, and lower-body muscular function using a leg press machine. Secondary outcomes will include upper-body muscular function, physical and cardiorespiratory function, blood pressure and heart rate, body composition, health-related quality of life, cognitive performance, anxiety, depression, physical activity levels, sleep and sedentarism, biochemical markers, motivators and barriers to exercise. Assessments will be conducted at baseline, mid-intervention (*i.e.,* week 13), at the end of the intervention (*i.e.,* week 25), and 24 weeks later (*i.e.,* week 49).

**Discussion:**

The findings of this trial might provide valuable insights into the role of supervision and motivational strategies on the effectiveness of exercise programs for older adults. Additionally, the study could contribute to developing cost-effective interventions, supporting the design of future public policies for healthy aging.

**Trial registration:**

NCT05619250. Registered 16 November 2022.

**Supplementary Information:**

The online version contains supplementary material available at 10.1186/s12877-024-04868-8.

## Background

Population aging is a highly prevalent phenomenon worldwide. Projections indicate that the global population aged 60 and above is expected to increase by more than 3 times, reaching nearly 2 billion by 2050 [1]. Aging is linked to physiological and functional impairments that can contribute to an increased risk of frailty, disability, and falls, increasing the susceptibility to chronic diseases [2–5]. It is well known that physical activity is an effective factor in reducing the risk of all-cause mortality and preventing the loss of intrinsic capacity (*i.e.,* the combination of all physical and mental capacities of a person) during aging [6–9]. There is strong evidence showing that regular exercise reduces age-related physical and mental decline [10, 11]. Specifically, multicomponent exercise interventions appear as an effective strategy for improving major outcomes such as muscle strength and mass or gait ability, as well as for reducing the incidence of falls and attenuating functional impairments [12–15]. Nevertheless, a large proportion of the population remains physically inactive. Particularly, a population-based study showed that only 15% of men and 10% of women aged ≥ 70 years achieved physical activity guidelines [16].

Although supervised center-based exercise interventions (herein referred to as supervised exercise for simplicity) are commonly prescribed to older adults, on some occasions feasibility can be limited by economic constraints, convenience, and access or time commitments [17, 18]. Under this context, unsupervised home-based exercise programs (herein referred to as unsupervised exercise) emerge as a potential alternative, and indeed different meta-analyses have shown that, compared with no exercise, unsupervised exercise can be effective for improving components of health and fitness in older adults [19, 20]. However, to date, there is no consensus on whether unsupervised exercise programs can be as effective –or even more cost-effective– than supervised ones. Some studies have shown greater benefits on different health variables with professionally supervised exercise programs compared to those performed autonomously at home [21–23]. On the other hand, other studies indicate that unsupervised exercise programs can be as effective as supervised ones [24–26]. For instance, Fisher et al. [27] found that supervised resistance exercise induces slightly greater muscle strength gains compared to unsupervised exercise in teenagers and adults, with minimal to no extra benefits on body composition. If their safety and cost-effectiveness are confirmed, unsupervised exercise programs would represent an alternative for increasing the accessibility of exercise for older adults, which would be of relevance for future public health policies. Nonetheless, most studies comparing supervised and unsupervised exercise have applied different interventions in each group (*e.g.,* different training types, duration, intensity, and exercise selection) [21]. Additionally, groups are often labeled as ‘supervised’ despite including some unsupervised sessions, and vice versa [28]. Therefore, well-designed randomized controlled trials (RCTs) that equalize exercise dose (*i.e.,* type, frequency, intensity, and volume) between both groups are warranted to draw definite conclusions on the role of supervision on the effectiveness of exercise programs.

One of the factors that could moderate the effectiveness of exercise programs is adherence. In this regard, a recent meta-analysis by our research group showed that adherence to unsupervised exercise programs is usually low [29], probably due to a lack of motivation. Applying motivational strategies might help increase adherence to exercise programs, which could eventually result in greater training-induced adaptations [30–32]. Some studies have analyzed the impact of motivational strategies during exercise programs in older adults [33, 34]. However, these studies analyzed the influence of motivational strategies in supervised or unsupervised exercise interventions separately, but never before have they been examined in a single study.

The Promoting Training Programs for Health (PRO-Training) trial aims to cover all the abovementioned research gaps. The main goal of this RCT will be to determine the impact of supervision and motivational strategies on the safety, adherence, efficacy, and cost-effectiveness of different exercise programs for improving physical and mental health in older people.

## Methods

**Fig. 1:**
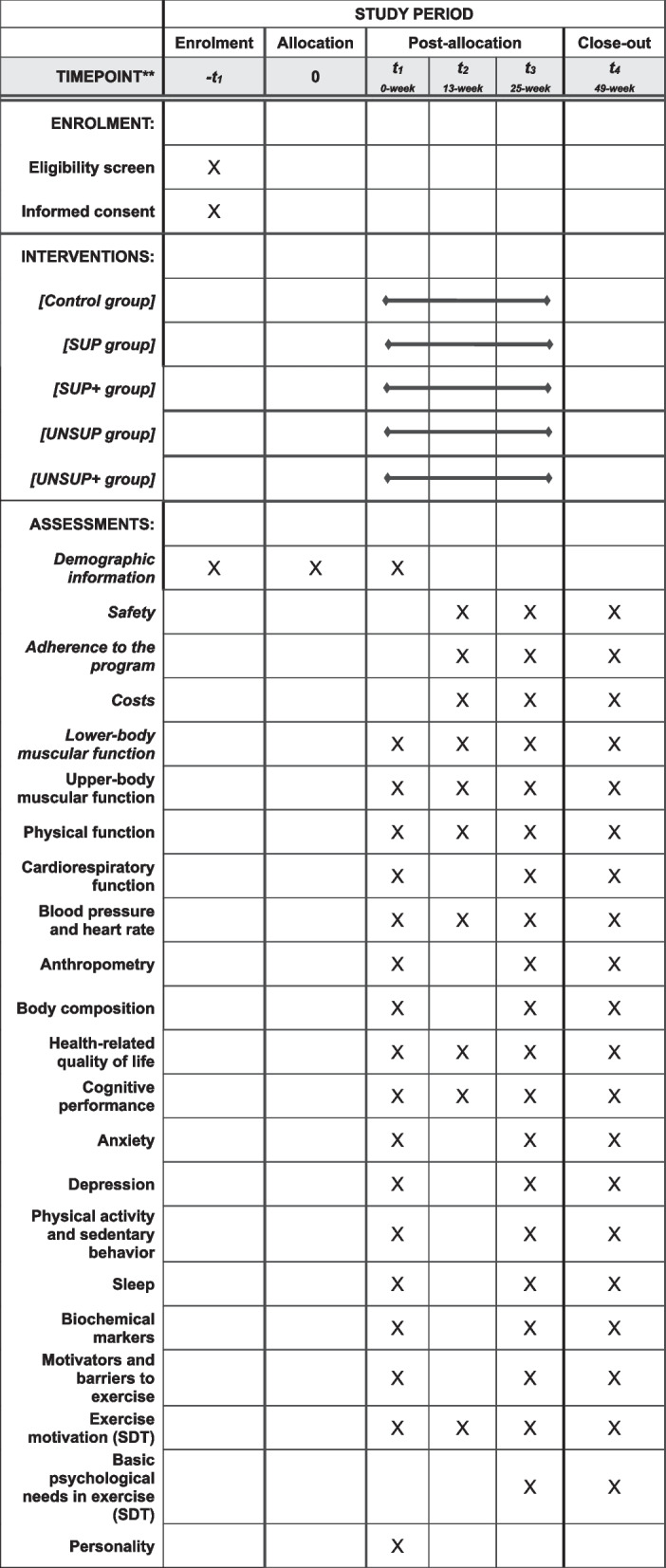
SPIRIT flow chart of study procedures. SDT: Self-Determination Theory; SUP: Supervised exercise without motivational intervention; SUP + : Supervised exercise with motivational intervention; -T1: enrolment of participants before assessments; T1: baseline assessment at 0-week; T2: mid-intervention assessment at 13-week; T3: post-intervention assessment at 25-week; T4: follow-up assessment at 49-week; UNSUP: Unsupervised exercise without motivational intervention; UNSUP + : Unsupervised exercise with motivational intervention

This protocol is reported according to the Standard Protocol Items: Recommendations for Interventional Trials (SPIRIT statement) [35] to ensure that all relevant information is included. A checklist is provided including this information (see Supplementary Table 1). The participant timeline recommended by SPIRIT includes the schedule of enrolment, interventions, and assessments (Fig. 1).

### Study design

**Fig. 2:**
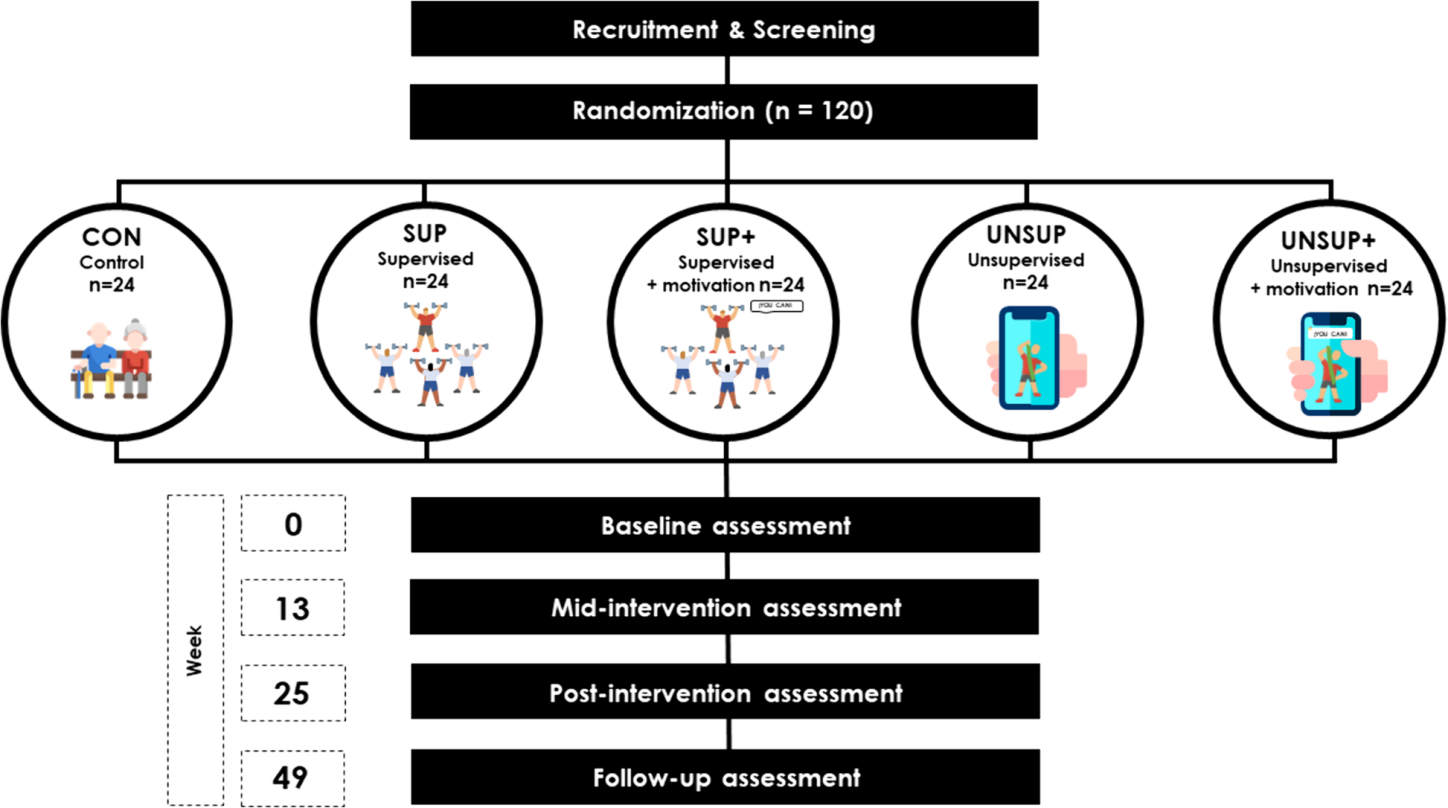
Flowchart of the study protocol. CON: Control group, SUP: Supervised exercise without motivational intervention, SUP + : Supervised exercise with motivational intervention, UNSUP: Unsupervised exercise without motivational intervention, UNSUP + : Unsupervised exercise with motivational intervention

The study will follow a single-blind randomized, parallel design. Participants will be randomized into 5 groups (Fig. 2): 1- Control group (CON), 2- Supervised exercise without motivational intervention (SUP), 3- Supervised exercise with motivational intervention (SUP +), 4- Unsupervised exercise without motivational intervention (UNSUP) and 5- Unsupervised exercise with motivational intervention (UNSUP +). Assessments will be conducted at baseline (week 0), mid-intervention (week 13), at the end of the intervention period (week 25), and 24 weeks later (week 49).

The study will be conducted at the University of Castilla-La Mancha (Spain). It will be reported in accordance with the Consolidation Standards of Reporting Trials (CONSORT) statements for cluster RCTs and Standard Protocol Items: Recommendations for Interventional Trials [36].

### Sample size

The sample size was determined by a priori power analysis using G ∗ Power 3.1.9.4 software. Input parameters were entered considering repeated measures ANOVA (group by time interaction), setting the probability of α error at 0.05, 1-β = 0.80, and 5 groups. The standardized mean difference used for calculations was 0.43, based on the effect of home-based exercise on lower-body muscle power in older adults reported in a previous meta-analysis [19]. The model indicated a total sample size of 100 individuals, and considering a dropout rate of 20%, we finally decided to enroll 120 participants.

### Recruitment

The trial will recruit a total of 120 participants through local, media, and social advertisements, posters, and informative talks. All participants will be informed about the purpose, procedures, benefits, and risks associated with participation in the study before signing a written consent form.

### Participant inclusion and exclusion criteria

The inclusion criteria will be the following: (1) individuals aged 60 to 75 years old; (2) fluency in Spanish language (both written and spoken); (3) ability to walk independently; (4) physical capability to engage in an exercise program; (5) competence in using a smartphone and mobile applications without assistance.

Participants meeting any of the following criteria will be excluded from the study: (1) individuals with a history of acute or terminal illness, as well as those who have experienced myocardial infarction, coronary artery bypass grafting, angioplasty, angina, or other cardiac conditions within the past year; (2) uncontrolled medical problems that the physician believes would preclude them from participating in an exercise program, such as acute systemic illness (*e.g.,* pneumonia), acute rheumatoid arthritis, and acute or unstable heart failure; (3) conditions requiring a specialized exercise program such as uncontrolled epilepsy, significant neurological disease or impairment, inability to maintain an upright seated position or unable to move independently, multiple sclerosis, cancer, Parkinson’s, Alzheimer’s, or chronic obstructive pulmonary disease; (4) uncontrolled hypertension diagnosed by a physician; (5) type I diabetes or uncontrolled type II diabetes; (6) history of major psychiatric illness (*e.g.,* schizophrenia, generalized anxiety disorder, or depression according to the DSM-5); (7) morbid obesity (body mass index > 39.9); (8) having experienced three or more self-reported falls in the last year; (9) not living in the community (i.*e.,* residing in nursing homes); (10) participation in an exercise program within the preceding six months; (11) having been diagnosed with COVID-19 with hospitalization in intensive care unit; (12) any other condition that could interfere with the study purposes and pose a risk to the participant, as determined by the researcher and physician. Eligibility criteria will be verified by a physician and a member of the research team during telephone screening and in the initial assessment. A medical history and the PAR-Q (Physical Activity Readiness Questionnaire) will be administered at the initial assessment.

Criteria for discontinuing intervention for a given trial participant include participant request, disease onset or worsening impairing research results or affecting exercise capacity, etc.

### Randomization and blinding

The randomization will be performed by a researcher not involved in the study. The allocation sequence will be determined by a computer-generated list (http://www.randomizer.org/). If a couple living in the same house participates, both will be randomized into the same group [37]. Blinding of the intervention is impossible for participants and therapists administering the intervention given its nature (*i.e*., exercise training). During the intervention, subjects will not be told about the existence of groups with and without motivational strategies. Statisticians and assessors in charge of outcome assessments will be blinded to participants’ allocation.

### Trial groups

All participants allocated to exercise intervention groups will complete a 24-week multicomponent exercise program consisting mostly of resistance exercises (3 sessions per week; 60 min per session). The exercise program will be reported following the Consensus on Exercise Reporting Template (CERT) [38] (see Supplementary Table 2). After completing the baseline assessment, participants will be randomly allocated to the 5 study groups. After randomization and irrespective of intervention group allocation, a member research team member will inform the participant about the intervention and assessment plan.

The exercise program for all intervention groups will be divided into 3 difficulty levels, increasing every 8 weeks. Each will include 3 sessions comprising of warm-up, balance, resistance, aerobic, and flexibility exercises (Tables 1 and 2). Further information about the training variables is available in Supplementary Table 3.

**Table 1:** Supervised exercise program

	**Session nº**	**Exercise nº**	**Intensity report**	**LEVEL 1**	**LEVEL 2**	**LEVEL 3**	**Type**
WARM-UP	1, 2 & 3	1	-	Seated ankle circumduction			Joint mobility
		2	-	Knee flexo-extension			
		3	-	Hip circumduction			
		4	-	Forward–backward shoulder circumduction			
		5	-	Wrist circumduction			
		6	-	Neck mobility			
		7	-	Diagonal foot touches	Foot touches forward, to the side and back with hands on the waist	Foot touches forward, to the side and back with arms in cross position	Balance
		8	-	Heel lifts	One leg balance	One leg balance with arms in cross position	
		9	-	Lateral movement without impact	Lateral movement with impact	Rope skipping	Soft cardio
		10	-	´V´ front	Knee-elbow	Low skipping	
MAIN ACTIVITY	1	1	-	One leg balance with arms in cross position	One leg balance with leg abduction	One leg balance with shoulder flexion	Balance
		2	RIR	Machine chest press	Cable crossover	Dumbbell chest press	UB push (pectoral, triceps)
		3	RIR	Box squat	Goblet squat	Jump squat	LB (quadriceps, gluteus)
		4	RIR	Pulley shoulder flys	Alternating dumbbells shoulder flys	Simultaneous dumbbells shoulder flys	UB pull (trapezius, deltoid)
		5	RIR	Assisted TRX lunge	Lunge	Weighted lunge	LB (quadriceps, gluteus)
		6	RPE	Front plank with shoulder touches	Front plank with knees	Front plank	Core
		7	RIR	Seated rowing machine	Standing cable row (medium height)	Unilateral dumbbell row	UB pull (dorsal)
		8	RIR	Knee-banded seated hip abduction	Monster walk	Clamshell	LB (gluteus)
		9	RPE	Lateral movements	Jumping jacks	Side-to-side shuffle	Cardio
		10	RPE	Bike or elliptical	Step machine	Running	
	2	1	-	Foot touches forward, to the side and back with hands on the waist	Foot touches forward, to the side and back with arms in cross position	Foot touches forward, to the side and back with arm movements at the same level	Balance
		2	RIR	Seated dumbbell shoulder press	TRX pendulum press	Standing barbell shoulder press	UB push (deltoid)
		3	RPE	Bird dog	Unilateral bird dog	Alternating bird dog with abduction	Core
		4	RIR	Pulley facepull	Pulley facepull with external rotation	Assisted scapular pull up	UB pull (trapezium, rhomboids)
		5	RIR	Leg press with external foot rotation	Multipower sumo squat	Weighted sumo squat	LB (quadriceps, gluteus)
		6	RPE	Pulley lateral rotation	Dynamic kneeling side plank	Static kneeling side plank	Core
		7	RIR	Standing cable row (high pulley)	Standing unilateral cable row (high pulley)	Standing cable pullover (high pulley)	UB pull (dorsal)
		8	RIR	Glute bridge	Hip thrust	Unilateral hip thrust	LB (gluteus)
		9	RPE	Slow climber	Beginner burpee	Beginner burpee with jump	Cardio
		10	RPE	Bike or elliptical	Step machine	Running	
	3	1	-	One leg balance with knee elevation	One leg balance with knee elevation and external rotation	One leg balance with hip extension and external rotation	Balance
		2	RIR	Pulley triceps extension	Dumbbell triceps kickback	Unilateral overhead triceps extension	UB push (triceps)
		3	RIR	Step up (alternative)	Step up (same leg)	Bulgarian squat with support	LB (quadriceps, gluteus)
		4	RIR	Pull down	Unilateral pull down	Machine assisted pull up	UB pull (dorsal, biceps)
		5	RPE	Isometric wall squat	Dynamic unilateral isometric wall squat	Static unilateral isometric wall squat	LB (quadriceps)
		6	RPE	Press pallof with elastic	Press pallof with elastic and disc	Pulley pallof press	Core
		7	RIR	Standing biceps cable curl	Alternating standing dumbbell biceps curl	Simultaneous standing dumbbell biceps curl	UB pull (biceps)
		8	RIR	Pull throught	Kettlebell deadlift	Barbell deadlift	LB (quadriceps, gluteus)
		9	RPE	Low skipping	Medium skipping	High skipping	Cardio
		10	RPE	Bike or elliptical	Step machine	Running	
COOL-DOWN	1, 2 & 3	1	-	Calf stretch	Dorsal stretch	Back stretch	Flexibility
		2	-	Chair hamstring stretch	Biceps and pectoral stretch	Pectoral stretch on the floor	
		3	-	Gluteus stretch	Deltoid stretch	Hamstring stretch with elastic	
		4	-	Quadriceps stretch with support	Lying quadriceps stretch	Psoas stretch	
		5	-	Back stretch with support	Standing hamstring stretch	Soleus stretch	
		6	-	Pectoral wall stretch	Neck stretch	Forearm stretch	
		7	-	Neck stretch	Adductor stretch	Seated gluteus stretch	

*LB* Lower-body, *RIR* Repetitions in reserve, *RPE* Rate of perceived exertion, *UB* Upper-body

**Table 2:** Unsupervised exercise program

	**Session nº**	**Exercise nº**	**Intensity report**	**LEVEL 1**	**LEVEL 2**	**LEVEL 3**	**Type**
WARM-UP	1, 2 & 3	1	-	Seated ankle circumduction			Joint mobility
		2	-	Knee flexo-extension			
		3	-	Hip circumduction			
		4	-	Forward–backward shoulder circumduction			
		5	-	Wrist circumduction			
		6	-	Neck mobility			
		7	-	Diagonal foot touches	Foot touches forward, to the side and back with hands on the waist	Foot touches forward, to the side and back with arms in cross position	Balance
		8	-	Heel lifts	One leg balance	One leg balance with arms in cross position	
		9	-	Lateral movement without impact	Lateral movement with impact	Rope skipping	Soft cardio
		10	-	´V´ front	Knee-elbow	Low skipping	
MAIN ACTIVITY	1	1	-	One leg balance with arms in cross position	One leg balance with leg abduction	One leg balance with shoulder flexion	Balance
		2	RIR	Wall push-ups	Quadruped push-ups	Knee push-ups	UB push (pectoral, triceps)
		3	RIR	Chair squat	Squat with elastic	Jumping squat	LB (quadriceps, gluteus)
		4	RIR	Vertical rowing with elastic	Alternating shoulder flys with elastic	Simultaneous shoulder flys with elastic	UB pull (trapezius, deltoid)
		5	RIR	Lunge with support	Lunge	Lunge with elastic	LB (quadriceps, gluteus)
		6	RPE	Front plank with shoulder touches	Front plank with knees	Front plank	Core
		7	RIR	Seated rowing with elastic	Simultaneous standing rowing with elastic	Unilateral standing rowing with elastic	UB pull (dorsal)
		8	RIR	Knee-banded seated hip abduction	Monster walk	Clamshell	LB (gluteus)
		9	RPE	Lateral movements	Jumping jacks	Side-to-side shuffle	Cardio
		10	RPE	Kneeling on the spot	Alternating step-up	Simultaneous step-up	
	2	1	-	Foot touches forward, to the side and back with hands on the waist	Foot touches forward, to the side and back with arms in cross position	Foot touches forward, to the side and back with arm movements at the same level	Balance
		2	RIR	Seated shoulder press with elastic	Unilateral seated shoulder press with elastic	Standing shoulder press with elastic	UB push (deltoid)
		3	RPE	Bird dog	Unilateral bird dog	Alternating bird dog with abduction	Core
		4	RIR	Scapular retraction with elastic	Alternating scapular retraction with elastic	Scapular retraction lying with elastic	UB pull (trapezium, rhomboids)
		5	RIR	Sumo chair squat	Sumo squat	Sumo squat with elastic band	LB (quadriceps, gluteus)
		6	RPE	Kneeling side plank	Kneeling side plank with leg abduction	Lateral rotation with elastic	Core
		7	RIR	Seated rowing with elastic	Simultaneous standing rowing with elastic	Unilateral standing rowing with elastic	UB pull (dorsal)
		8	RIR	Glute bridge with elastic	Frog pump with elastic	Unilateral glute bridge with elastic	LB (gluteus)
		9	RPE	Slow climber	Beginner burpee	Beginner burpee with jump	Cardio
		10	RPE	Kneeling on the spot	Alternating step-up	Simultaneous step-up	
	3	1	-	One leg balance with knee elevation	One leg balance with knee elevation and external rotation	One leg balance with hip extension and external rotation	Balance
		2	RIR	Seated triceps extension with elastic	Triceps kickback with elastic	Unilateral overhead triceps extension with elastic	UB push (triceps)
		3	RIR	Lunge with support	Lunge	Lunge with elastic	LB (quadriceps, gluteus)
		4	RIR	Scapular retraction with elastic	Pull with elastic	Alternating pull with elastic	UB pull (dorsal, biceps)
		5	RPE	Isometric wall squat	Dynamic unilateral isometric wall squat	Static unilateral isometric wall squat	LB (quadriceps)
		6	RPE	Dead bug with leg movements	Dynamic dead bug	Static dead bug	Core
		7	RIR	Seated biceps curl with elastic	Alternating standing biceps curl with elastic	Simultaneous standing biceps curl with elastic	UB pull (biceps)
		8	RIR	Sumo deadlift with elastic	Conventional deadlift with elastic	Unilateral conventional deadlift with elastic	LB (quadriceps, gluteus)
		9	RPE	Low skipping	Medium skipping	High skipping	Cardio
		10	RPE	Lateral movements	Jumping jacks	Side-to-side shuffle	
COOL-DOWN	1, 2 & 3	1	-	Calf stretch	Dorsal stretch	Back stretch	Flexibility
		2	-	Chair hamstring stretch	Biceps and pectoral stretch	Pectoral stretch on the floor	
		3	-	Gluteus stretch	Deltoid stretch	Hamstring stretch with elastic band	
		4	-	Supported quadriceps stretch	Lying quadriceps stretch	Psoas stretch	
		5	-	Back stretch with support	Standing hamstring stretch	Soleus stretch	
		6	-	Pectoral wall stretch	Neck stretch	Forearm stretch	
		7	-	Neck stretch	Adductor stretch	Seated gluteus stretch	

*LB* Lower-body, *RIR* Repetitions in reserve, *RPE* Rate of perceived exertion, *UB* Upper-body

- *1- Control group (CON)*

Participants will not perform any other exercise program during the intervention period and will be advised to maintain their usual lifestyle.

- *2- Supervised exercise without motivational intervention (SUP)*

The SUP group will participate in an exercise program conducted at university facilities in small groups (≤ 8 participants per session) under the supervision of a sports scientist. The program will be conducted with the equipment usually available at a gym (*i.e.,* weight machines, dumbbells, kettlebells, discs, pulleys, elastic bands, and stationary bicycles).

- *3- Supervised exercise with motivational intervention (SUP +)*

The SUP + group will receive the same exercise program as the SUP group (Table 1) but with motivational strategies based on self-determination theory designed to increase participants’ perception of autonomy, competence, and relatedness. These strategies will be based on 6 colors simulating martial arts levels, from white belt (beginner) to black belt (advanced). Each level will employ different strategies to achieve the specific objectives set for that level. Motivational strategies include telephone calls, educational infographics, workshops and videos about exercise benefits, motivational messages before and after each training session, and reports of the results obtained in the mid-intervention assessment. More details about the motivational strategies are available in Supplementary Table 4.

- *4- Unsupervised exercise without motivational intervention (UNSUP)*

The UNSUP group will complete an unsupervised exercise program prescribed via a mobile app designed for at-home use without face-to-face supervision. The program will involve exercises that target the same muscle groups and movements as the supervised groups but are adapted to be performed at home using elastic bands and participants’ body weight.

- *5- Unsupervised exercise with motivational intervention (UNSUP +)*

The UNSUP + group will receive the same exercise program as the UNSUP group (as detailed in Table 2), plus equivalent motivational strategies to those administered to the SUP + group. More information is available in Supplementary Table 4.

At the end of the 24-week intervention, all participants (including those in the CON group) will be provided with elastic bands and a list of training centers supervised by sports scientists (including a discount on some of them). Thus, they will have equal opportunities to engage in the exercise practice that best suits their circumstances, preferences, tastes, and interests.

### Outcome measures

#### Primary outcomes

##### Safety

The number of falls and adverse events will be recorded. Participants in the CON, UNSUP, and UNSUP + groups will record their falls and adverse events during and outside exercise sessions with a diary [39, 40]. In the SUP and SUP + groups, the sports scientist will register the incidence of falls and adverse events during exercise sessions, and participants will register them outside the exercise sessions using a diary.

##### Adherence to the program

Adherence to the program will be calculated as a ratio between the sessions completed from those initially prescribed ([sessions completed/total sessions expected] × 100), where 0% indicates total non-adherence and 100% indicates full adherence [40]. The sports scientist will record adherence to the exercise program in the SUP and SUP + groups [41], while in the UNSUP and UNSUP + groups, it will be automatically registered through the mobile app [42].

##### Costs

Total costs and costs per participant will be calculated for each intervention group. The mean difference in quality-adjusted life-year (QALY) scores at the end of the intervention will be assessed to analyze the cost-utility. QALY scores range from 1 (perfect health) to 0 (dead) [43]. Furthermore, an incremental cost-effectiveness ratio (ICER) will be calculated by dividing the adjusted cost differences by the effect differences. Data on health-related quality of life measured through the EuroQoL five dimensions (EQ-5D) questionnaire will be used for the calculations [44–47].

##### Lower-body muscular function

The force–velocity relationship and the rate of force development (RFD) will be assessed unilaterally on a horizontal leg press machine (Selection MED, Technogym, Italy) instrumented with a force plate (Type 9286BA, Kistler, Switzerland) and a linear position transducer (TForce System, Ergotech, Spain). A familiarization session will be performed on the first day of assessments. During the RFD test, participants will receive instructions to execute multiple maximal voluntary isometric contractions. They will be required to rapidly and forcefully extend their knee while maintaining the contraction for 3 s following the cue *"Ready, set, go!"* [48].

Four adequate trials (avoiding countermovement before contraction onset), separated by 60 s will be acquired alternatively (knee angles of 90º and 120º). The maximal isometric force and RFD will be calculated as the linear slope of the time-force curve over different time intervals (0–400 ms) and at the maximal point of the time-RFD curve (RFDmax) [49]. To assess the force–velocity relationship, participants will perform a progressive loading test (5–20 kg increments) with 2 repetitions per load (leaving a few seconds between repetitions to ensure peak performance on the subsequent repetition), starting at approximately 40% of the individual one repetition maximum (1-RM) (corresponding to the first load in which there is no flight phase) until reaching a load equivalent to 80% 1-RM. Loads will be interspersed by 1-min of passive recovery. From the starting position (90º knee angle), participants will be instructed to perform the concentric phase of each repetition as fast and as strong as possible, regardless of the load used. The values of force and velocity of the most powerful repetitions with each load will be fitted by a linear model through the Excel spreadsheet of Alcazar et al. [50] from which we will estimate the theoretical maximal isometric force and the maximal velocity without load (intercepts of force and velocity, respectively). In addition, we will estimate the maximum muscular power as well as the force and velocity at which it is produced [51].

#### Secondary outcomes

##### Upper-body muscular function

Handgrip strength will be assessed on both arms using a digital hand-held dynamometer (Takei TKK5401, Tokyo, Japan), with the handle individually adjusted to fit each participant´s hand. Participants will be seated in a chair with their arm extended and forearm in a neutral position, holding the dynamometer in a vertical position. Before performing the test, the assessor will demonstrate and hand over the dynamometer to the participant for familiarization [52]. After the starting cue, the subject will be encouraged to squeeze as strongly as possible during a 3-s isometric contraction without additional body movement. There will be a 1-min rest between each repetition. The same procedure will be repeated for the opposite hand. Two attempts will be performed, and the maximum value will be chosen.

##### Physical function

Firstly, the Senior Fitness Test (SFT) will be conducted, which includes 6 functional tests: (1) 30-s chair stand test for lower-limb strength, (2) 30-s arm curl test with a 2.5-kg or 4-kg dumbbell for upper-limb strength, (3) 6-min walk test for aerobic endurance, (4) chair sit-and-reach test for lower-limb flexibility, (5) back scratch test for upper-limb flexibility and (6) 8-foot up-and-go test for agility/dynamic balance [37, 53]. Secondly, the Short Physical Performance Battery (SPPB) will be performed to evaluate static balance in feet side-by-side, semi-tandem, and tandem positions, 4-m-usual walking speed, and 5-repetition sit-to-stand test [54]. Additionally, the maximum speed when walking 10 m [55, 56] and the usual speed when walking 3, 6, and 10 m [57] will be also examined. All tests will be performed once, except the walking tests, which will be performed twice. The best value will be chosen in the maximum speed test and the average in the usual walking speed tests.

##### Cardiorespiratory function

A graded exercise test (GXT) and a verification test (VerT) will be conducted using an incremental and a supramaximal test, respectively [58]. Both tests will be performed on an electrically braked cycle ergometer (800S, Ergoline, Bitz, Germany). Oxygen consumption and carbon dioxide production will be evaluated breath by breath using indirect calorimetry (Quark RMR, COSMED, Rome, Italy). A standard 12-lead electrocardiogram (12SL-ECG, GEhealthcare, Finland) will be performed at rest and during exercise to assess the heart’s electrical activity. For the GXT, after a 3-min warm-up at 15W for men and 10W for women, the workload will be increased 1W/7 s for women (8.5W/minute) and 1W/5 s for men (12W/minute) until volitional exhaustion. These increment rates have been designed to bring subjects to exhaustion in 8 to 12 min [59]. Subjects will receive verbal encouragement to reach their maximum effort. Upon reaching exhaustion, the workload will decrease to 10W, and active recovery will be performed by pedaling for 3 min. After 10 min of passive seated rest, participants will complete a constant-load VerT, which consists of a 1-min warm-up at 50% of the maximal workload achieved during the GXT (Wmax),followed by an increase in workload up to 110% Wmax [58]. Verbal encouragement will be given to achieve maximum pedaling time to exhaustion. The test will end when the cycling cadence drops below 45 rpm for 10 consecutive seconds.

##### Blood pressure and heart rate

Resting blood pressure and heart rate will be assessed using a digital upper-arm blood pressure monitor (OMRON M6 Comfort, Omron Healthcare, Kyoto, Japan) [60]. Subjects will be asked to sit in a quiet room for 10 min before the assessments. The selection of cuff size will be chosen according to the left arm circumference of each participant; the blood pressure cuff will be placed on bare skin. Blood pressure will be assessed in a seated position at the level of the right atrium, with the participant’s back supported and uncrossed legs with both feet on the floor. A second separated measurement will be done after a 5-min seated rest period [61].

##### Anthropometry

Subjects’ height and body mass will be measured with a stadiometer and scale device (Seca 711, Hamburg, Germany) to the nearest 0.1 cm and 0.1 kg, respectively. The subject will be barefoot and wearing as minimal clothes as possible. The neck will be kept in a natural unstretched position, with the heels touching each other with the fingertips separated to form a 45° angle and the head held straight with the lower orbital rim in the Frankfort plane [62]. Body mass index will be calculated as body mass (kg) divided by height (m) squared (kg·m^−2^). In addition, the perimeters of the neck, waist, and hip will be measured [63, 64]. The neck circumference will be assessed by placing the tape measure around the neck below the Adam's apple. Waist circumference will be measured by positioning the tape at the midpoint of the last palpable rib and the top of the hip bone. For hip circumference, the measuring tape will be wrapped around the maximum circumference of the buttocks and below the iliac crest. The subject will be asked to inhale air and perform the measurement at the end of a normal exhalation.

##### Body composition

For the determination of bone mass and body composition, 4 densitometry scans will be performed on each subject using a calibrated dual-energy X-ray absorptiometry (DXA, Hologic Series Horizon-A, Bedford, United States). Lean mass (kg), fat mass (kg), bone mineral content (BMC, g), and bone mineral density (BMD, g·cm^−2^) will be assessed in the total and appendicular analysis of the whole-body scan. In addition, BMC and BMD will be assessed in the non-dominant forearm, the proximal region of the right femur (total hip, femoral neck, intertrochanter, greater trochanter, and Ward’s triangle), and the lumbar spine (L1–L4). Assessments will be performed with participants in light clothing, barefoot, and without jewellery or removable metal. All DXA scans will be analyzed using a specific software (Physician’s Viewer, APEX System Software v.5.6.1.3, Hologic, Bedford, MA, United States).

##### Health-related quality of life

The EQ-5D questionnaire will be used for assessing participants’ health status, including a descriptive system formed by 5 dimensions and a visual analog scale (EQ-VAS) [65]. The 5 dimensions included in the descriptive system are: (1) mobility, (2) self-care, (3) usual activities, (4) pain/discomfort, and (5) anxiety/depression. The EQ-VAS scale consists of requesting participants to indicate their current health status from 0 ("*worst imaginable health status*") to 100 ("*best imaginable health status*").

##### Cognitive performance

The Trail-Making Test is a neuropsychological test with two parts [66]. In part A, the participant must connect the numbers from 1 to 25 in numerical order, while in part B, the subject must connect the dots in order while alternating letters (from A to L) and numbers (1 to 13), as quickly as possible without lifting the pencil from the paper. The Montreal Cognitive Assessment (MoCA, available at www.mocatest.org) is a single-page 30-item test completed in approximately 10 min that assesses visuospatial abilities, identification, memory, attention, language, 2-item verbal abstraction, delayed recall and orientation to time and place [67]. The Digit Symbol Substitution test is a pencil-and-paper psychomotor performance test [68] that requires the subject to match symbols to numbers according to a key located at the top of the page. The score is the number of right combinations of numbers and symbols that can be achieved in 2 min [69, 70].

##### Anxiety

The Zung Anxiety Self-Assessment Scale comprises 20 items related to anxiety symptoms (both psychological and somatic) [71]. Responses will be reported on a 4-point scale ranging from 1 (none or rarely) to 4 (most or all of the time).

##### Depression

The short form of the Yesavage Geriatric Depression Scale has proven to be a valid instrument for screening depression in older people [72]. This scale consists of 15 items with a yes/no answer pattern. Ten items show the presence of depression when the answer is positive (numbers 2, 3, 4, 6, 8, 9, 9, 10, 12, 14, 15), while the rest indicate depression when the answer is negative [73].

##### Physical activity and sedentary behavior

Physical activity levels and sedentary patterns will be assessed for 8 consecutive days using accelerometry (GeneActiv Original, Activinsights, UK) [74, 75]. The devices will be set to record data at 60 Hz and will be placed on the participant's non-dominant wrist. Additionally, they will receive a diary log to record when they remove the accelerometer. Furthermore, self-reported physical activity and time spent sitting will be measured using the International Physical Activity Questionnaire Short Form (IPAQ-SF) [76], which is a 7-item self-report questionnaire that calculates daily metabolic equivalents (METs) for the last 7 days, including vigorous activities, moderate activities, walking and sitting time. During the 48-week follow-up, an ad-hoc self-reported questionnaire will be used to assess questions related to exercise practice (see Supplementary File 1). The questionnaire will include the following questions: (1) *“Have you exercised on a regular basis during the last 6 months (from the post-intervention assessments until now)?”,* (2) *“What type(s) of exercise have you done during the last 6 months?”, (3) “What average of days a week have you exercised regularly?”, (4) “What average time (hours and/or minutes) have you exercised each day on a regular basis?”, (5) “What average intensity is best associated with the exercise you have done on a regular basis?”, (6) “Have you ever exercised under the supervision of a professional?”, (7) “Where have you usually exercised during the last 6 months?”, (8) “What type of equipment have you usually used for training during the last 6 months?”* and *(9) “Have you used any type of motivational strategy during the last 6 months?”*.

##### Sleep

The number of sleeping hours will be measured by accelerometry (GeneActiv Original, Activinsights, UK) for 8 consecutive nights, in addition to filling out a sleep diary to report nightly sleep periods [75, 77]. Additionally, the Pittsburgh Sleep Quality Index (PSQI) will be used to measure sleep quality during the last month [78]. The PSQI consists of 19 self-rated questions grouped into 7 component scores, including subjective sleep quality, sleep latency, sleep duration, habitual sleep efficiency, sleep disturbances, use of sleeping medications, and daytime dysfunction.

##### Biochemical markers

Blood samples will be obtained from the antecubital vein after an overnight fast of at least 12 h. Participants should refrain from moderate-to-vigorous physical activity for at least 72 h before the blood test. Analyses will include complete blood cell count, chemistry panel, lipid profile, electrolytes, and creatinine kinase. Samples will be analyzed immediately for routine clinical chemistry measurements and then placed in 500-µL aliquots to be frozen at -80ºC for further analysis.

##### Motivators and barriers to exercise

Participants’ perceived motivators and barriers to exercise will be assessed through the Benefits/Barriers to Exercise Scale [79], a 43-item instrument with a four-response Likert-type format: (1) strongly disagree, (2) disagree, (3) agree, and (4) strongly agree. Twenty-nine items are related to the benefits scale, while the remaining 14 are barriers. The total score of the instrument can range from 43 to 172 points, with higher scores indicating a more positive perception of exercise.

##### Motivation to exercise according to the self-determination theory

The Behavioral Regulation during Exercise Questionnaire (BREQ-3) [80, 81] comprises 23 items (4 for intrinsic regulation, 4 for integrated regulation, 3 for identified regulation, 4 for introjected regulation, 4 for external regulation and 4 for discouragement) that measure the stages of the self-determination theory with respect to motivation to exercise. Participants will answer each item on a 5-point scale ranging from 0 (not true for me) to 4 (very true for me).

##### Basic psychological needs in exercise

The Basic Psychological Needs in Exercise Scale (BPNES) will be used to measure the basic psychological needs of the participants during exercise. It is a 5-point scale: (1) I don’t agree at all, (2) I agree a little bit, (3) I somewhat agree, (4) I agree a lot, and (5) I completely agree. This scale has previously proven to be a valid instrument for assessing the 3 needs of autonomy, competence, and relatedness in the exercise domain according to the self-determination theory [82, 83].

##### Personality

Personality will be assessed using the Spanish version of the NEO-Five Factor Inventory (NEO-FFI) [84, 85]. This questionnaire measures the Big Five personality factors (*i.e.,* neuroticism, extraversion, openness, conscientiousness, and agreeableness) through 60 items on a 5-point Likert scale: (1) I strongly disagree, (2) disagree a little, (3) neither agree nor disagree, (4) agree a little and (5) strongly agree [86].

##### Confounding variables

Confounding variables will be assessed in baseline using an ad-hoc demographic questionnaire, including the following: (1) sex (*i.e.,* male or female); (2) birth date; (3) marital status (*i.e.,* single, married, widowed, divorced, or domestic partner); (4) living situation (*i.e.*, living alone, with partner or with family); (5) educational level (*i.e.,* elementary, high school or university); (6) socioeconomic level (*i.e.,* < 14.000, 14.000–30.000 or > 30.000 euros per year); (7) race (*i.e.,* caucasian, african-american, asian, native-american or other); (8) smoking status (*i.e.,* non-smoker, ex-smoker [not having smoked in the last 6–12 months] or current smoker); (9) self-reported falls in the last year (*i.e.,* none, one or two); (10) number of diseases (*i.e.,* none, cardiovascular disease, cerebrovascular disease, high blood pressure, anxiety and/or depression, diabetes mellitus, high cholesterol, osteoporosis, renal disease, liver disease, cirrhosis, hepatic insufficiency, osteoarthrosis, rheumatoid arthritis, hyperthyroidism, hypothyroidism or other); (11) alcohol consumption (*i.e.,* never, once a month or less, 2–4 times a month, 2–3 times a week, 4 or more times a week) and (12) body mass index. These data will be used for descriptive statistics and as covariates for the analyses.

### Data collection and management

**Fig. 3:**
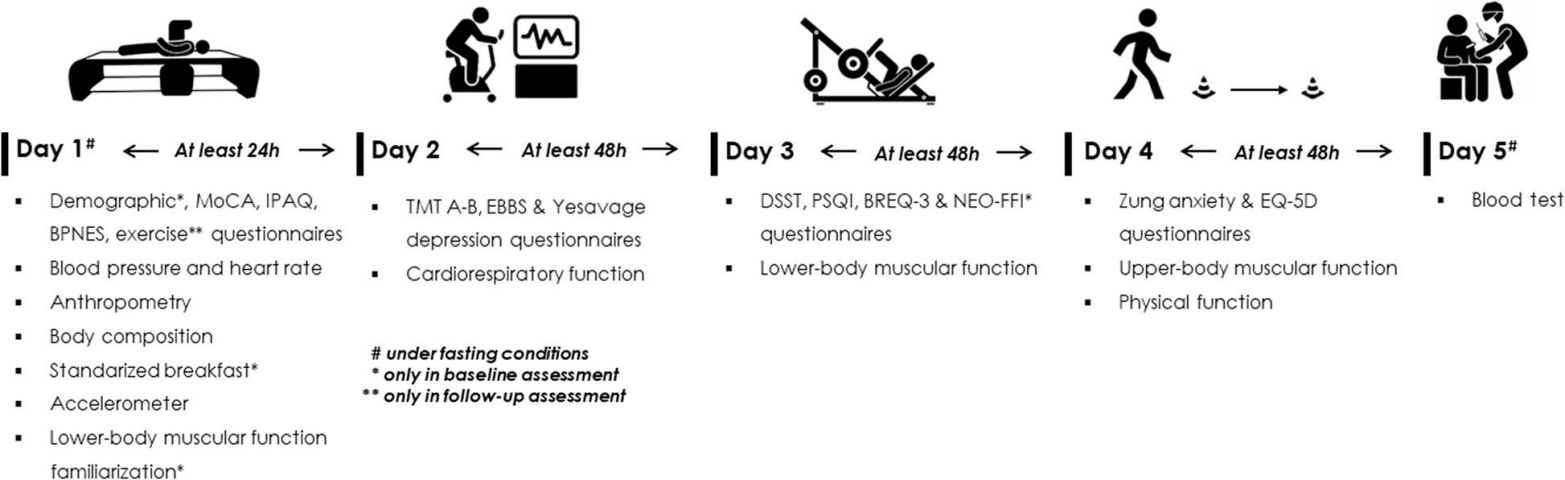
Assessment protocol. BPNES: Basic Psychological Needs in Exercise Scale; BREQ-3: Behavioral Regulation during Exercise Questionnaire; DSST: Digit Symbol Substitution Test; EBBS: Exercise Benefits/Barriers Scale; EQ-5D: EuroQoL-5 dimensions quality of life questionnaire; IPAQ: International Physical Activity Questionnaire; MoCA: Montreal Cognitive Assessment; NEO-FFI: NEO-Five Factor Inventory questionnaire; PSQI: Pittsburgh Sleep Quality Questionnaire; TMT A-B: Trail-Making Test Part A and B

Outcomes will be assessed at baseline, mid-intervention (*i.e.,* week 13), at the end of the intervention (*i.e.,* week 25), and 24 weeks later (*i.e.,* week 49). At each measurement point –except for the mid-intervention measurement, which will only involve 1 session– participants must perform all assessments in 5 different days interspersed by at least 48 h and a maximum of 1 week. Trained assessors will conduct all assessments. A detailed scheme of the outcomes assessed in this study is shown in Figs. 1 and 3.

Participation in the project is voluntary, so participants may withdraw from the study at any time. Data collected from them are retained confidentially by the project team and included in the analyses. Personal information about potential and enrolled participants will be collected, shared, and maintained with codes to protect confidentiality throughout the trial.

The paper data will be stored in coded (re-identifiable) format in a locked filing system at the University of Castilla-La Mancha. The electronic data will be encrypted (re-identifiable) and stored in private computer systems. Only the research team will have access to the trial data. The databases will be prepared by two independent evaluators with double data entry.

### Statistical analysis

Data will be preferentially presented as mean ± standard deviation or as proportions for continuous and categorical variables, respectively. Data normality will be assessed by the Kolmogorov–Smirnov test and will be log-transformed in case of non-normal distribution. Baseline differences will be assessed through a one-way ANOVA. A two-way mixed ANCOVA (group [*i.e.*, CON, SUP, SUP + , UNSUP, and UNSUP +] x time [*i.e.,* baseline, mid, post-intervention, and follow-up assessments]) will be performed, adjusting for baseline values as well as for any other sociodemographic/descriptive variables that may differ between groups (*e.g.,* age, sex). Subsequent post-hoc tests will be conducted to determine which groups significantly differ. Effect sizes will be calculated to assess the magnitude of the differences [87]. Falls and fall-related injuries will be presented as rates/person (ITT analysis) and rates/person/year (± 30 days; PP analysis), and as numbers. Adverse events and dropouts will be compared between groups through Chi-squared tests. Differences in adherence rates will be analyzed using an ANOVA test. Correlation analyses will be used to assess associations between motivators, barriers, and adherence to exercise sessions. Whenever possible, statistical analyses will be performed on an intention-to-treat (ITT) basis (including all randomized participants). The potential effects of missing values and dropouts will also be explored using multiple imputation and sensitivity analyses [88]. As recommended, a complementary per protocol (PP) analysis will be performed (*i.e.,* including only those participants with valid data for baseline and post-intervention assessments and an adherence to exercise program of at least 80%) [89]. SPSS version 24.0 (IBM Corp., Armonk, NY) will be used for statistical analysis, and the significance level will be set at p ≤ 0.05 for all tests.

### Ethics and dissemination

The implementation of the activities foreseen in this project will be in accordance with international principles and current regulations on bioethics, biosafety, biosecurity, environmental protection, natural heritage and biodiversity, cultural heritage, gender equality, and data protection, and respect the fundamental principles set out in the Declaration of Helsinki (World Medical Assembly). The dissemination of results will not be subject to any restrictions. It is expected that the study findings will be published and presented through a variety of channels, including peer-reviewed journal publications, conference presentations, posters or oral communications within exercise industry, and social media. Anonymity of participants will be guaranteed.

### Trial status

This trial is currently active. The first participant was recruited on 7 September 2022. It is anticipated that recruitment will be completed in late 2024.

## Discussion

The main objective of the present trial is to examine the role of supervision and motivational strategies on the safety, adherence, efficacy, and cost-effectiveness of 4 different exercise programs for improving physical and mental health among people between 60 and 75 years old. We hypothesize that all exercise programs will be safe, and those that add cognitive-behavioral and motivational strategies will result in greater adherence and efficacy. Furthermore, we expect that UNSUP + will be the most cost-effective option, thereby increasing the accessibility of exercise to all older adults and consequently improving their health and quality of life.

As recommended by the World Health Organization, older adults should perform 150–300 min/week of moderate-intensity aerobic physical activity, 75–150 min/week of vigorous-intensity aerobic physical activity, or a combination thereof, as well as multicomponent physical activity focusing on functional balance and resistance training (≥ 3 days per week) [90]. The benefits of multicomponent exercise for mental and physical health are widely known [91–94]. Regular exercise helps in the prevention and management of different chronic diseases (*e.g.*, diabetes, obesity or overweight, hypertension, osteoporosis, or osteopenia) [95–98], thus leading to reduced attendance to medical consultations. According to Santos et al. [99], the global cost of inaction on physical inactivity would cost healthcare systems approximately 47.6 billion $ per year. If we add to this the low participation of older adults in physical activity, which decreases with age, we justify the need for safe and effective exercise programs for this population [16, 100].

Studying the influence of supervision and motivation during exercise programs could allow us to know which exercise interventions are more profitable, safer and result in higher adherence rates for this population. The recent COVID-19 pandemic has shown that face-to-face exercise is not always feasible, highlighting the relevance of alternative strategies when mobility restrictions or accessibility difficulties exist [101]. Home-based exercise sessions have increased in popularity during the pandemic [102–104]. In this sense, the development of the mobile application used by the unsupervised exercise groups in the present study might provide a tool with scientific validity to all those older adults who want to start an exercise program from home. This technological tool could be useful for people who are physically or financially limited, have reduced time, or perceive a certain feeling of rejection to exercise in a sports center. It could also be helpful for all those who live in unpopulated rural areas and do not have access to a facility or sports center where they can exercise. Furthermore, motivational strategies might be particularly helpful in this population because of the low rate of participation in exercise programs [29], and they are not usually familiar with digital technologies [105, 106].

Different RCTs have previously compared the effectiveness of SUP *versus* UNSUP [22, 107]. However, to date, a shared limitation has been a reduced duration of exercise programs (usually < 10 weeks) [108, 109], so in the present study, the intervention will last 24 weeks. Another relevant limitation in prior research has been the unequal type of exercise intervention between SUP and UNSUP. In the present trial, the exercise program of UNSUP and UNSUP + groups is designed to be as similar to SUP and SUP + as possible but adapted so that every participant can exercise without requiring expensive material. Furthermore, previously published studies have not accounted for the potential influence of motivational strategies [33].

This project displays considerable ambition in its design. Consequently, some critical risks related to its implementation and possible sources of bias require attention. Firstly, there may be variation in the outcome measures obtained if certain standardized conditions are not followed. To this end, participants will be given the preconditions for each session (*e.g.,* fasting, clothing). A minimum of 24 h will be left between one evaluation session and another if tests requiring physical effort are involved, as muscle damage may distort the results of other evaluations. Secondly, there may be some bias in the measurement of different outcomes according to the assessor performing them. Therefore, evaluators have received specialized training and standardized protocols have been designed to ensure consistency of measurements to the greatest extent possible. Furthermore, evaluators will be blinded, so that those administering the exercise program will only be able to participate in the baseline assessments to reduce bias. Lastly, the recruitment of the planned sample may be challenging. To address this problem, we plan to recruit participants from previous studies and to use a variety of recruitment channels. In addition, if the desired sample size is not achieved, a second wave of recruitment may be conducted.

**Fig. 4:**
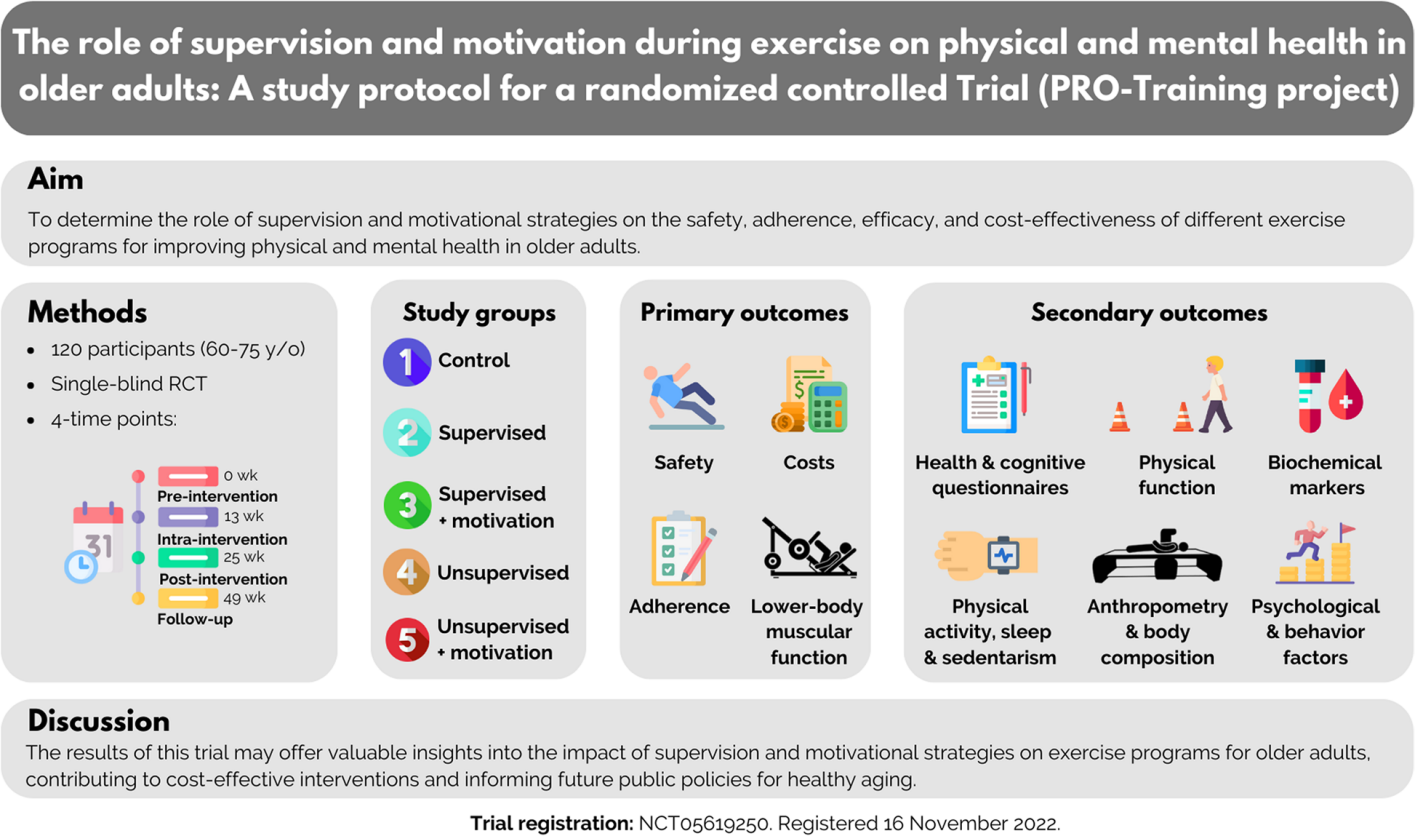
Graphical abstract of the PRO-Training project. RCT: Randomized Controlled Trial; wk: week; y/o: years old

To the best of our knowledge, this will be the first RCT that analyzes the influence of motivational strategies by equating the training dose of SUP and UNSUP groups in older adults for 24 weeks. Therefore, our results could complement trials on the influence of supervision and the inclusion of motivational strategies in exercise programs designed to improve the health of older people feasibly and cost-effectively. In conclusion, the findings of the PRO-Training project might shed light on the impact of supervision and motivational strategies during a 24-week multicomponent training program on the physical and mental health of older adults (see in Fig. 4). Therefore, we hope this project represents a scientific advance, responding to the detected gaps in the literature. Moreover, we hope this project will provide meaningful benefits not only for the study participants but also for the health systems, being able to help implement evidence-based policies aimed at improving the well-being of older adults.

### Supplementary Information

**Supplementary Material 1.**

**Supplementary Material 2.**

**Supplementary Material 3.**

**Supplementary Material 4.**

**Supplementary Material 5.**

## Data Availability

All data used for this analysis can be acquired at any time by contacting the corresponding author upon reasonable request.

## Abbreviations

BMC: Bone mineral content
BMD: Bone mineral density
BPNES: The basic psychological needs in exercise scale
BREQ-3: Behavioral regulation during exercise questionnaire
CERT: Consensus on exercise reporting template
CONSORT: Consolidation standards of reporting trials
CON: Control group
DSM-5: Diagnostic and statistical manual of mental disorders, fifth edition
DXA: Dual-energy X-ray absorptiometry
EQ-5D: EuroQoL five dimensions questionnaire
EQ-VAS: Visual analogue scale
GXT: Graded exercise test
ICER: Incremental cost-effectiveness ratio
IPAQ-SF: International physical activity questionnaire short form
ITT: Intention-to-treat
MoCA: Montreal cognitive assessment
PAR-Q: Physical activity readiness questionnaire
PP: Per protocol
PSQI: Pittsburgh sleep quality index
QALY: Quality-adjusted life-year
RCT: Randomized controlled trial
RFD: Rate of force development
SFT: Senior fitness test
SPPB: Short physical performance battery
SUP: Supervised exercise without motivational intervention
SUP +: Supervised exercise with motivational intervention
UNSUP: Unsupervised exercise without motivational intervention
UNSUP +: Unsupervised exercise with motivational intervention
VerT: Verification test
