# Supplementary material for: The role of supervision and motivation during exercise on physical and mental health in older adults: a study protocol for a randomized controlled trial (PRO-Training project)
Source: BMC Geriatr. 2024 Mar 20;24:274. doi: 10.1186/s12877-024-04868-8 (PMC10953175; doi:10.1186/s12877-024-04868-8)
Supplement: Supplementary file 2 — Supplementary Material 2. [file 12877_2024_4868_MOESM2_ESM.doc]

| Supplementary Table 2. Consensus on Exercise Reporting Template (CERT) checklist. | | | | |  | |
| --- | --- | --- | --- | --- | --- | --- |
|  | **Item** | **Description** | **Groups** | | | |
|  |  |  | **SUP** | **SUP+** | **UNSUP** | **UNSUP+** |
| WHAT: materials  Exercise equipment | 1 | Detailed description of the type of exercise equipment. | Equipment available at the facility: guided weight machines, free weights (*i.e*., dumbbells, discs, and barbells), cardio machines (*i.e.*, exercise bike, stair climber and elliptical). | | Auxiliary furniture materials, mobile application, and elastic bands of 6 different resistance levels. | |
| WHO: provider  Instructor qualifications, experience &/or training | 2 | Detailed description of the qualifications, expertise and/or training. | Sports scientist. | | | |
| HOW: delivery  Individual or group exercise | 3 | Describe whether exercises are performed individually or in a group. | Small groups of up to 8 people. | | Autonomously and individually supported by a mobile application specifically designed for this purpose. | |
| Supervised or unsupervised & mode of delivery | 4 | Describe whether exercises are supervised or unsupervised; how they are delivered. | Supervised presencially by a sports scientist. | | Unsupervised, performed at each participant's home. | |
| Exercise adherence measurement & reporting | 5 | Detailed description of how adherence to exercise is measured and reported. | The sports scientist will register the attendance of the participants to group classes. | | The mobile application will automatically register the number of workouts completed by the participants. | |
| Motivation strategies | 6 | Detailed description of motivation strategies. | N/A. | Based on Self-Determination Theory (Supplementary Table 4). | N/A. | Based on Self-Determination Theory (Supplementary Table 4). |
| Decision rules for exercise progression | 7a | Detailed description of the decision rule(s) for determining  exercise progression. | The overload principle will be applied throughout the 6-month intervention with the aim of increasing the difficulty of the workouts, through:   - Intensity: - *RIR:* it changes for each level, depending on the first or last weeks of training (see progression in Supplementary Table 3). - *External resistance:* participants must reach the proposed RIR in the execution time set, adjusting the perceived intensity with the different elastic bands available. - Time: execution time changes with each level, depending on the first weeks of training or the last weeks of training. - Type: - *Exercise selection:* the same structure of the sessions will be maintained based on the type of exercise for the 4 training groups. Based on this, exercises with similar movement patterns were selected considering the peculiarities of the equipment available at the facility (gym or home) (see Tables 1 and 2). - *Execution type:*    - Concentric phase: as fast as possible.   - Eccentric phase: controlled. | | | |
| How exercises were progressed | 7b | Detailed description of how the exercise program was progressed. |  |  |  |  |
| Detailed description of each exercise | 8 | Detailed description of each exercise to enable replication. | Exercise selection is shown in Table 1. | | Exercise selection is shown in Table 2. | |
| Home program component | 9 | Detailed description of any home program component. | N/A. | | Mobile application that provides a workout log (*i.e.*, RIR achieved) and training guide. | |
| Non-exercise components | 10 | Describe whether there are any non-exercise components. | N/A. | | Mobile application. | |
| Type and number of adverse events during exercise | 11 | Describe the type and number of adverse events that occur during exercise. | The expected risks during the training are low and infrequent. During the first sessions muscle soreness ("stiffness") may be experienced late in the musculature involved. Adverse events that could result from this study are mainly related to the practice of exercise: falls, cardiovascular problems, fatigue, muscle injury, dizziness, or fainting, etc. | | | |
|  |  |  | Participants will register falls and/or adverse events in and out the training session using a logbook provided for that purpose. At the same time, researchers will note those reported through phone calls, messages or in assessments. | | | |
| WHERE: location  Setting in which exercises are performed | 12 | Describe the setting in which the exercises are performed. | Gym located in the Campus Fábrica de Armas (Toledo, Spain). | | Home of each participant. | |
| WHEN, HOW MUCH: dosage  Detailed description of exercise intervention | 13 | Detailed description of the exercise intervention. | See Table 1 and Supplementary Tables 2 and 3. | | See Table 2 and Supplementary Tables 2 and 3. | |
| TAILORING: what, how  Generic or individually tailored | 14a | Describe whether the exercises are generic (one size fits all) or tailored. | The individualization principle will be applied to adapt the training program as follows:   - A gradual progression of the selected exercises will be applied over the course of the intervention, involving the same muscles with similar movement patterns. Thus, exercises are selected to be familiar to participants so that emphasis can be placed on the acquisition of good technique during their execution. - Exercises will be executed at the maximum possible speed during the concentric phase. - Each participant must perform as many repetitions as they can according to the target RIR during the execution time. - Participants will select the number and type of elastic band needed to reach the target RIR based on their perceived exertion. | | | |
| How exercises were individually tailored | 14b | Detailed description of how exercises are tailored to the individual. |  |  |  |  |
| Decision rule for exercise starting level | 15 | Describe the decision rule for determining the starting level. | It will be the same for all participants and will be adapted by the sports scientist if necessary. | | It will be the same for all participants. | |
| HOW WELL: planned, actual   - Measurement of adherence to the intended intervention - Actual adherence intended intervention | 16a | Describe how adherence or fidelity is assessed/measured. | The sports scientist will register the attendance of the participants using a checklist. | | A register will be kept using the mobile application. | |
|  | 16b | Describe the extent to which the intervention was delivered as planned. | N/A. | | | |

N/A: Not available; RIR: Repetitions in reserve; SUP: Supervised exercise without motivational intervention; SUP+: Supervised exercise with motivational intervention; UNSUP: Unsupervised exercise without motivational intervention; UNSUP+: Unsupervised exercise with motivational intervention.
