# Supplementary material for: The role of supervision and motivation during exercise on physical and mental health in older adults: a study protocol for a randomized controlled trial (PRO-Training project)
Source: BMC Geriatr. 2024 Mar 20;24:274. doi: 10.1186/s12877-024-04868-8 (PMC10953175; doi:10.1186/s12877-024-04868-8)
Supplement: Supplementary file 3 — Supplementary Material 3. [file 12877_2024_4868_MOESM3_ESM.doc]

**Supplementary Table 3.** Training variables.

|  |  | | LEVEL 1 | | | LEVEL 2 | | | LEVEL 3 | | |
| --- | --- | --- | --- | --- | --- | --- | --- | --- | --- | --- | --- |
|  | | | **Months 1 and 2** | | | **Months 3 and 4** | | | **Months 5 and 6** | | |
| Frequency | Times/week | | 3 days per week | | | | | | | | |
| Duration | Weeks | | *Weeks 1-2* | *Weeks 3-5* | *Weeks 6-8* | *Weeks 9-10* | *Weeks 11-13* | *Weeks 14-16* | *Weeks 17-18* | *Weeks 19-21* | *Weeks 22-24* |
| Intensity | *Resistance* | RIR target *(concentric-eccentric exercises)* | 4 - 6 | 2 - 4 | 2 - 4 | 4 - 6 | 2 – 4 | 2 – 4 | 4 - 6 | 2 – 4 | 2 – 4 |
|  |  | RPE target  *(isometric exercises)* | 4 - 6 | 6 - 8 | 6 - 8 | 4 - 6 | 6 - 8 | 6 - 8 | 4 - 6 | 6 - 8 | 6 - 8 |
|  |  | External resistance (*e.g.*, elastic band, weight) | To be selected by the participants based on their perception of effort.  The sports scientist will prescribe external resistance in SUP and SUP+ groups. | | | | | | | | |
|  | *Aerobic* | RPE target | 6 - 7 | 7 - 8 | 7 - 8 | 6 - 7 | 7 - 8 | 7 - 8 | 6 - 7 | 7 - 8 | 7 - 8 |
| Time | *Balance, resistance & aerobic* | Execution time | 30 seconds | 40 seconds | 50 seconds | 30  seconds | 40  seconds | 50  seconds | 30  seconds | 40  seconds | 50  seconds |
|  |  | Resting time | 60 seconds | | | | | | | | |
|  | *Warm-up & flexibility* | Execution time | 15 seconds | | | | | | | | |
|  |  | Resting time | 10 seconds | | | | | | | | |
| Volume | Sets | | 3 sets per exercise | | | | | | | | |
|  | Repetitions | | Maximum number of repetitions achieved according to the target RIR during execution time *(Not for isometric exercises)* | | | | | | | | |
| Type | *Exercise* | Multicomponent:   - Warm-up: joint mobility, balance, and soft cardio. - Main part: 1 balance exercise, 7 resistance exercises, 2 aerobic exercises. - Cool-down: 7 flexibility exercises. | | | | | | | | | |
|  | *Execution* | Concentric phase: as fast as possible.  Eccentric phase: controlled.  Technical/security: follow the instructions detailed by the sports scientist or the mobile application. | | | | | | | | | |

RIR: repetitions in reserve; RPE: rate of perceived exertion; SUP: supervised exercise without motivational intervention; SUP+: supervised exercise with motivational intervention.
