# Supplementary material for: The role of supervision and motivation during exercise on physical and mental health in older adults: a study protocol for a randomized controlled trial (PRO-Training project)
Source: BMC Geriatr. 2024 Mar 20;24:274. doi: 10.1186/s12877-024-04868-8 (PMC10953175; doi:10.1186/s12877-024-04868-8)
Supplement: Supplementary file 4 — Supplementary Material 4. [file 12877_2024_4868_MOESM4_ESM.doc]

| **Supplementary Table 4.** Motivational strategies based on Self-determination Theory. | | | | | | | |
| --- | --- | --- | --- | --- | --- | --- | --- |
|  | **MOTIVATIONAL STRATEGIES BY LEVELS** | | | | | | |
|  | **LEVEL 1 (BASIC)** | | **LEVEL 2 (INTERMEDIATE)** | | **LEVEL 3 (ADVANCED)** | | **End of 24-week intervention** |
|  | **LEVEL OF SELF-DETERMINATION**  **↑** | | | | | |  |
|  | **WHITE BELT**  **↓** | **YELLOW BELT** | **ORANGE BELT** | **GREEN BELT** | **BLUE BELT** | **BROWN BELT** | **BLACK BELT** |
| **STEP TO THE NEXT LEVEL** | At least **4 weeks** from the start of the program.  At least **50% sessions (≥ 6 sessions)** with white level. | At least **8 weeks** from the start of the program.  At least **50% sessions (≥ 6 sessions)** with yellow level. | At least **12 weeks** from the start of the program.  At least **65% sessions (≥ 8 sessions)** with orange level. | At least **16 weeks** from the start of the program.  At least **65% sessions (≥ 8 sessions)** with green level. | At least **20 weeks** from the start of the program.  At least **80% sessions (≥ 10 sessions)** with blue level. | At least **24 weeks** from the start of the program.  At least **80% sessions (≥ 10 sessions)** with brown level. |  |
| **OBJECTIVES** | 1. To experience satisfaction from initiating a program to enhance physical fitness and health, understanding its benefits. 2. To be able to start the training program. | 1. To progressively overcome additional barriers related to physical exercise every day. 2. To feel satisfaction upon having consistently completed a month of training. 3. To experience satisfaction in completing the same training with reduced fatigue compared to its initial stages. 4. To enjoy the benefits of improved physical abilities and health. | 1. To experience satisfaction from establishing a training routine. 2. To achieve adherence successfully. | To recognize individual improvement from the outset, considering aspects of physical condition (in comparison to normative values for one's age) and other associated benefits (such as an enhanced quality of life). | To feel competition and enjoyment. | To self-regulate their motivation and be able to train on their own. |  |
| **IMPLEMENTATION** | White | Yellow | Orange | Green | Blue | Brown | Black |
| - **SUP+:**  belt color wristband. - **UNSUP+** mobile app screen based on belt color. |  |  |  |  |  |  |  |
| - **SUP+:** on-site at the Campus Fábrica de Armas. - **UNSUP+:** by video call. | Initial workshop: project purpose and exercise benefits (recommendations, etc.). |  |  | Intermediate workshop: providing strategies and feedback to address key exercise-related barriers. |  | Final workshop: discussion and strategies for achieving consistency in long-term training to sustain adherence during follow-up. |  |
| - **SUP+ and UNSUP+:**  telephone call. | 1 weekly call. | 1 biweekly call | 1 biweekly call | 1 biweekly call | 1 monthly call | 1 monthly call |  |
| - **SUP+:**  will be sent via an individual chat. - **UNSUP+:** the mobile app shows the infographic and video. | 1 infographic:   - Exercise benefits.   1 video:   - Exercise benefits. | 2 infographics:   - WHO physical activity guidelines.   1 video:   - Relevance of meeting WHO physical activity guidelines. | 5 infographics:   - Mortality risk due to physical inactivity.   1 video:   - Healthy aging. | 6 infographics:   - Myths related to exercise. | 4 infographics:   - Knowledge of the muscle groups used during the exercise program.   5 infographics:   - Integration of learned exercises into daily life. | 6 infographics:   - Knowledge of the type of exercise performed during exercise sessions.   2 videos:   - Discipline. |  |
| - **SUP+:**  will be sent via an individual WhatsApp chat prior to the training session. - **UNSUP+:** the mobile app shows a pre-workout phrase. | **Week 1:**   1. Thank you for choosing this program designed for seniors who want to improve their physical condition and well-being. 2. Today we do a basic level training, let's go for it! 3. As you will see, each workout consists of 10 exercises in its main part. We will always maintain that structure.   **Week 2:**   1. Today we are going to repeat last week's training, to see how we are able to do it. 2. Today we are going to focus on performing the exercises with proper technique to avoid injuries. 3. Let's enjoy moving.   **Week 3:**   1. We are going to increase the intensity of the training by increasing the duration of the exercises, are you up for it? 2. Today's training is the same as two weeks ago. Do we try to do more repetitions of each exercise? 3. Thank you for prioritizing your health and organizing your time to do your training today.   **Week 4:**   1. *“Setting objectives is the first step to transform the invisible into visible”*Cheer up with your training today! 2. We are going to increase the intensity of the training by decreasing the RER, are you up for it? 3. We are going to increase the intensity of the training by increasing the duration of the exercises, are you up for it? | **Week 5:**   1. Once the first month of adaptation is over, we will try to do this session with a little more intensity. 2. Today we do a basic level training, let's go for it! 3. On the same structure of 10 exercises, today we will try to increase the number of repetitions.   **Week 6:**   1. Today we are going to try to perfect the technique of the exercises. 2. To make training more difficult, we will do the same exercises as last week, but for longer. 3. Today we are going to enjoy getting in shape.   **Week 7:**   1. Thank you for training one more day: it is noticeable that you have more and more strategies to avoid missing your health appointment. 2. Today's training is the same as two weeks ago. Do we try to do more reps on each set? 3. Thanks for getting inwalk one more day, little by little you will get into the habit, and it will be easier.   **Week 8:**   1. This training, in addition to being fun, improves your health. 2. As they say “Rome was not built in a day”. Be persevering with training and you will be able to reach your best version! 3. Feel satisfied that you are improving your physical abilities and health. Let's go for training! | **Week 9:**   1. You have already completed 2 months of training and you are getting better every time, let's go for more! 2. Today we do an intermediate level training, let's go for it! 3. Convince your mind so that your body can give more of itself in each exercise. With a good state of mind and attitude you will get a good workout.   **Week 10:**   1. We continue with the objective of improving your physical condition and your health. 2. We are going to fight against physical inactivity by doing dynamic exercises where we spend most of the time in motion. 3. Today we will enjoy a session with a lot of movement.   **Week 11:**   1. We are going to increase the intensity of the training by decreasing the RER, are you up for it? 2. Congratulations on the regularity you are acquiring in training. Let's go for a demanding one. 3. Today's training is the same as two weeks ago. Do we try to do more repetitions of each exercise?   **Week 12:**   1. Today you don't have to think too much, just enjoy and let yourself go. 2. We are going to increase the intensity of the training by increasing the duration of the exercises, are you up for it? 3. Do you want to do a session that fills you with vitality? Go! | **Week 13:**   1. You have already reached green level: that means that you have already shown regularity in training for 3 months: congratulations. Let's go for today's training. 2. Today we do an intermediate level training, let's go for it! 3. On the same structure of 10 exercises, today we will try to increase the number of repetitions.   **Week 14:**   1. We are going to increase the intensity by increasing the duration of the exercises, are you up for it? 2. Congratulations on finding this time for yourself, your health and your enjoyment. 3. Today we will try to do the same circuit that we have done before, but with more load.   **Week 15:**   1. Have you thought about what your goal is for today? Improve your resistance? To be stronger? Let's go for him! 2. Today we are going to do an exercise session that you already know, but with more intensity. 3. Look back and think about everything you've achieved so far. Keep it up, you're amazing!   **Week 16:**   1. Strength and growth come from continuous effort. You are on the right track! 2. Don't wait for the right time to do things, the only time is NOW. You can with today's training! 3. Let's go with the last session of this intermediate level. | **Week 17:**   1. You have reached the blue level: congratulations for these 4 months improving your skills and enjoying moving your body. 2. Today we do an advanced level training, let's go for it! 3. Every time you can execute more complex exercises, keep it up!   **Week 18:**   1. After 4 months of training, your heart and muscles are working better and better. Let's exercise them! 2. Your current physical shape allows you to do more difficult exercises, let's go for them”. 3. Each training is a unique opportunity to improve. Let's go for today!   **Week 19:**   1. We are going to increase the intensity of the training by increasing the duration of the exercises, are you up for it? 2. With this type of sessions we will be able to improve our strength, resistance and balance to become more functional in the activities of daily life. 3. We are going to increase the intensity of the training by decreasing the RER, are you up for it?   **Week 20:**   1. Today's session requires you to be in shape as you already are and keep your attention to the maximum. 2. Thank you for being here, NOW is your time. Let's go for today's training! 3. Let's go with the last session of this month, you can! | **Week 21:**   1. You have already completed 5 months of training and have reached the advanced level. You must be very proud of what you are achieving! 2. Today we do a training of the most advanced level, let's go for it! 3. On the same structure of 10 exercises, today we will try to increase the number of repetitions.   **Week 22:**   1. We are going to increase the intensity by increasing the duration of the exercises, are you up for it? 2. Congratulations! Each time you are becoming a more autonomous person to train. 3. Each time you are able to do more demanding exercises. Congratulations!!   **Week 23:**   1. Are you looking forward to a new training session? Let's go for her!! 2. You are increasingly capable of training without external help. 3. Let's challenge ourselves.   **Week 24:**   1. We are going to enjoy a training session that some time ago would have been too much effort. 2. Today you will enjoy moving your body. 3. You have more and more independence to exercise. |  |
| - **SUP+:**  will be sent via an individual WhatsApp chat at the end of the training session. - **UNSUP+:** the mobile app shows a post-workout phrase. | **Week 1:**   1. We have already started. Congratulations on starting this exercise program to improve your health. 2. Thank you for your effort, I'm sure little by little you'll start to feel better training. 3. You have completed your first week of training – congratulations on GETTING ACTIVATED.   **Week 2:**   1. Thank you for choosing to train with us to improve your health. 2. With this training you have your dose of health for today. 3. Today you have managed to be a physically active person, congratulations.   **Week 3:**   1. Every day in the mirror you will see who you have to overcome. Congratulations on improving yourself one more day! 2. Feel satisfied for having added one more training! 3. Today you will go to bed with more satisfaction for having completed this training. Keep it up!   **Week 4:**   1. All achievement begins with the decision to try, and you are trying. Congratulations on completing this training! 2. REPEAT WITH ME “I am going to be 10 times better, in the next 6 months” You have completed your training! Keep it up! 3. Congratulations on completing your first month of training – we are well underway. | **Week 5:**   1. Congratulations on completing this most intense session. I knew you could handle her! 2. Do you remember when you did this training at the beginning of the program? I'm sure today turned out better. 3. I hope you enjoyed this training.   **Week 6:**   1. Thank you for your effort. 2. You sure have been able to train harder now than last month. 3. Undoubtedly, having chosen to train today will improve your abilities and your autonomy.   **Week 7:**   1. Thanks to your effort you have been able to perform these exercises that will improve your physical shape. 2. Today you have been able to overcome one more training session. Feel satisfied with your work! 3. Thank you for completing this workout which will help you improve your fitness level and health.   **Week 8:**   1. It is not about "having time", it is about "taking time" and today you have achieved it. Congratulations! 2. Training completed! Now a shower, a good hydration and enjoy with charged batteries. 3. Congratulations on completing 2 months of training! | **Week 9:**   1. You have endured the new exercises very well. You are a champion! 2. Your body is younger thanks to exercise. Congratulations on adding one more workout! 3. Every day you are able to do more demanding exercises.   **Week 10:**   1. Congratulations on your effort, your health and well-being have improved thanks to it. 2. Your legs and arms are getting stronger! 3. Remember, success and improvement begins at the end of your comfort zone. Congratulations on completing this training!   **Week 11:**   1. Our task in life is not to beat others, but to surpass ourselves. Congratulations on surpassing yourself one more day! 2. Take small actions daily that bring you a little closer to the results you want to achieve. Keep it up! 3. Rate your progress, it is better to progress slowly than not progress at all. One more workout that adds up!   **Week 12:**   1. *"Those who think they don't have time to exercise sooner or later will have to find time to cure their diseases."* 2. This session has been more intense, so CONGRATULATIONS ON COMPLETING IT! 3. CONGRATULATIONS! You have completed 3 months of training. You've passed the halfway point of the show! | **Week 13:**   1. Do you remember your sensations after training 3 months ago? What do you find better? The adaptation is noticeable. 2. You are gaining more and more autonomy, both for training and for your day-to-day life. 3. You are creating an excellent habit for health, congratulations.   **Week 14:**   1. I hope you enjoyed the training. Good job! 2. Each time you have a better level of physical condition. You are going for the good way! 3. Think that challenges are what make life interesting and overcoming them is what gives us meaning... You are on the right track!   **Week 15:**   1. *"You are what you do, not what you say you will do"*Congratulations on doing this workout! 2. Congratulations on completing this most intense session. I knew you could handle it! 3. Congratulations! You are progressing at an incredible rate, keep it up!   **Week 16:**   1. I hope you enjoyed the training. Good job! 2. After these weeks of training, we will be able to move to the next level, congratulations. 3. Congratulations on completing 4 months of training! | **Week 17:**   1. You have endured very well the incorporation of new exercises. Great! 2. Congratulations, you have completed advanced level training. 3. Thanks to your perseverance, you are improving your health and well-being.   **Week 18:**   1. Do you remember your fitness level 4 months ago? I'm sure you feel better now. 2. Surely you are enjoying the training more and more. 3. The harder you work, the harder it is to quit. Keep it up!   **Week 19:**   1. This session has been more intense, so CONGRATULATIONS ON COMPLETING IT! 2. How to reach a goal? Slowly but surely. Keep it up, you're doing great! 3. *"What separates the successful from the unsuccessful is perseverance"* Continue to persevere with your workouts!   **Week 20:**   1. With this training, you have improved your endurance and muscular strength. Congratulations! 2. You have more and more autonomy in your training. It’s amazing! 3. We like to know that you are enjoying more and more. | **Week 21:**   1. How do you think you would have done this session 5 months ago? I'm sure much better today. Congratulations! 2. It always seems impossible until it's done, and you've done it. Congratulations on completing this session! 3. Your physical condition is getting better, the effort made these months is noticeable.   **Week 22:**   1. Congratulations on completing this most intense session. It shows all the work of these months! 2. Congratulations, enjoy the feeling of a job well done. 3. Today's training was not easy, congratulations on finishing it!   **Week 23:**   1. The active lifestyle you lead now suits you very well. 2. *"MOTIVATION drives us to start and HABIT allows us to continue"* Keep it up! 3. *"You will not always be motivated, you have to learn to be disciplined "*Keep it up!   **Week 24:**   1. Feel proud @ of the path you're walking and how far you're getting. 2. The body is made to move and enjoy moving. We hope you have enjoyed. 3. Congratulations on completing these 6 months of training, YOU NOW HAVE THE CAPACITY TO TRAIN WITHOUT HELP! |  |
| - **SUP+ and UNSUP+:** will be sent by mail. |  |  |  | Brief report on the results obtained in the intra-intervention assessment. |  |  |  |

SUP: Supervised exercise without motivational intervention; SUP+: Supervised exercise with motivational intervention.

The colors are based on the martial arts levels from white belt (beginner) to black belt (advanced). It is used to structure participants motivation throughout the intervention. Thus, they can see their progress in a symbolic way from one color of belt to another until they reach the maximum level of autonomy.
