# Supplementary material for: The role of supervision and motivation during exercise on physical and mental health in older adults: a study protocol for a randomized controlled trial (PRO-Training project)
Source: BMC Geriatr. 2024 Mar 20;24:274. doi: 10.1186/s12877-024-04868-8 (PMC10953175; doi:10.1186/s12877-024-04868-8)
Supplement: Supplementary file 5 — Supplementary Material 5. [file 12877_2024_4868_MOESM5_ESM.pdf]

**Supplementary File 1. Follow-up exercise**

1. *Have you exercised on a regular basis during the last 6 months (from the post-intervention assessments until now)?*
   - Yes
   - No

2. *What type(s) of exercise have you done during the last 6 months? DO NOT INCLUDE WALKING. You can select more than 1 option.*
   - Resistance (*e.g.,* body weight, elastic bands, dumbbells)
   - Balance (*e.g.,* yoga, taichi)
   - Flexibility (*e.g.,* stretching, yoga, taichi)
   - Aerobic (*e.g.,* running, cycling, swimming)
   - Others:

3. *What is the average number of days per week that you have exercised on a regular basis? DO NOT INCLUDE WALKING.*
   - 1 day
   - 2 days
   - 3 days
   - 4 days
   - 5 days
   - 6 days
   - Every day

4. *What average time (hours and/or minutes) have you exercised each day on a regular basis?*
   - Free short answer:

5. *What average intensity is best associated with the exercise you have done on a regular basis?*
   - Light (it is practically effortless)
   - Moderate (you find it hard to maintain a conversation, but you are able)
   - Vigorous (you find it very difficult to maintain a conversation or you are unable to maintain a conversation)

6. Have you exercised under the supervision of a professional?
   - I have exercised under professional supervision
   - I have exercised without the supervision of a professional

7. *Where have you usually exercised during the last 6 months?*
   - Gym/sports center
   - Home
   - Outdoors (*e.g.,* parks, nature)

8. *What type of equipment have you usually used for training during the last 6 months? You can select more than 1 option.*
   - Body weight
   - Elastic bands
   - Guided machines

- Dumbbells
- Discs and bars

9. *Have you used any type of motivational strategy during the last 6 months?*
    - Yes
    - No

    If the answer is yes, describe the strategies used:

10. Other comments:
